# Supplementary material for: A Critical View of the Application of the APEX Software (Aqueous Photochemistry of Environmentally-Occurring Xenobiotics) to Predict Photoreaction Kinetics in Surface Freshwaters
Source: Molecules. 2019 Dec 18;25(1):9. doi: 10.3390/molecules25010009 (PMC7017383; doi:10.3390/molecules25010009)
Supplement: Supplementary file 1 [file molecules-25-00009-s001.zip › 3.2.4_gcc-4.4.0.zip!/3.2.4_gcc-4.4.0/3.2.4_gcc-4.4.0/doc/octave/html/interpreter/The-INDEX-file.html]

The INDEX file - Untitled


Next: PKG\_ADD and PKG\_DEL directives,
Previous: The DESCRIPTION File,
Up: Creating Packages


---

#### 35.4.2 The INDEX file

The optional `INDEX` file provides a categorical view of the
functions in the package. This file has a very simple format

- Lines beginning with # are comments.- The first non-comment line should look like this

    ```
              toolbox >> Toolbox name
    ```

    - Lines beginning with an alphabetical character indicates a new
      category of functions.- Lines starting with a white space character indicate that the
        function names on the line belong to the last mentioned category.

The format can be summarized with the following example

```
     # A comment
     toolbox >> Toolbox name
     Category Name 1
      function1 function2 function3
      function4
     Category Name 2
      function2 function5
```

If you wish to refer to a function that users might expect
to find in your package but is not there, providing a work around or
pointing out that the function is available elsewhere, you can use:

```
     fn = workaround description
```

This workaround description will not appear when listing functions in the
package with `pkg describe` but they will be published
in the html documentation online.
Workaround descriptions can use any html markup, but
keep in mind that it will be enclosed in a bold-italic environment.
For the special case of:

```
     fn = use <code>alternate expression</code>
```

the bold-italic is automatically suppressed. You will need
to use `<code>` even in references:

```
     fn = use <a href="someothersite.html"><code>fn</code></a>
```

Sometimes functions are only partially compatible, in which
case you can list the non-compatible cases separately. To
refer to another function in the package, use `<f>fn</f>`.
For example,

```
     eig (a, b) = use <f>qz</f>
```

Since sites may have many missing functions, you can define
a macro rather than typing the same link over and again.

```
     $id = expansion
```

defines the macro id. You can use `$id` anywhere in the
description and it will be expanded. For example,

```
     $TSA = see <a href="link_to_spctools">SPC Tools</a>
     arcov = $TSA <code>armcv</code>
```

id is any string of letters, numbers and `_`.
